# Who Is Right? Behavioral Problems from the Perspectives of Parents and Children with ADHD symptoms

**DOI:** 10.1192/j.eurpsy.2024.677

**Published:** 2024-08-27

**Authors:** K. Sitnik-Warchulska, B. Izydorczyk, I. Markevych, M. Szwed, A. Sawicki, M. Lipowska

**Affiliations:** ^1^ Institute of Applied Psychology; ^2^Institute of Psychology, Jagiellonian University, Krakow; ^3^Institute of Psychology, University of Gdansk, Gdansk, Poland

## Abstract

**Introduction:**

Diagnosing behavioral problems in children and adolescents, which include conduct symptoms, anxiety, or somatic complaints, is frequently based on subjective perceptions and interviews with family or caregivers. However, current theoreticians and practitioners of systemic theory are increasingly emphasizing that there are multiple subjective narratives about oneself, the world, and one’s symptoms. The question is whether these narratives are equivalent, and if not, under what circumstances do they diverge?

**Objectives:**

The study aimed to investigate whether the perception of behavioral problems among young adolescents with ADHD aligns with their parents’ perspective, and whether family bonding is a factor in this association.

**Methods:**

The analytic sample comprised about 200 children, aged 10-14 years, and their parents, mostly coming from well-situated families. The data were collected as a part of the NeuroSmog project. The variables were measured by the Child Behaviour Checklist (CBCL), the Youth Self Report (YSR), the Family Adaptation and Cohesion Evaluation Scales (FACES-IV). The structural equation modelling (SEM) to analyse data was used. The models were also stratified by age, sex, and social status.

**Results:**

There is a significant difference between the perspectives of parents and children regarding the level of behavioral problems. Family bonding is associated with behavioral problems among children, but this relationship is only evident from their perspective.

**Conclusions:**

The perception referring to family narratives has the most significant impact on individual functioning.

**Disclosure of Interest:**

None Declared

